# Contribution of Organically Grown Crops to Human Health

**DOI:** 10.3390/ijerph110403870

**Published:** 2014-04-08

**Authors:** Eva Johansson, Abrar Hussain, Ramune Kuktaite, Staffan C. Andersson, Marie E. Olsson

**Affiliations:** 1 Department of Plant Breeding, The Swedish University of Agricultural Sciences, P.O. Box 101, Alnarp, SE 23053, Sweden; E-Mails: Ramune.kuktaite@slu.se (R.K.); Staffan.andersson@slu.se (S.C.A.); Marie.olsson@slu.se (M.E.O.); 2 Department of Biosciences, COMSATS Institute of InformationTechnology, Sahiwal Campus, Comsats Road, Sahiwal 57000, Pakistan; E-Mail: Abrar.hussain@ciitsahiwal.edu.pk

**Keywords:** organic farming, micronutrients, bioactive compounds, *in vitro*, pesticide residues, animal studies

## Abstract

An increasing interest in organic agriculture for food production is seen throughout the world and one key reason for this interest is the assumption that organic food consumption is beneficial to public health. The present paper focuses on the background of organic agriculture, important public health related compounds from crop food and variations in the amount of health related compounds in crops. In addition, influence of organic farming on health related compounds, on pesticide residues and heavy metals in crops, and relations between organic food and health biomarkers as well as *in vitro* studies are also the focus of the present paper. Nutritionally beneficial compounds of highest relevance for public health were micronutrients, especially Fe and Zn, and bioactive compounds such as carotenoids (including pro-vitamin A compounds), tocopherols (including vitamin E) and phenolic compounds. Extremely large variations in the contents of these compounds were seen, depending on genotype, climate, environment, farming conditions, harvest time, and part of the crop. Highest amounts seen were related to the choice of genotype and were also increased by genetic modification of the crop. Organic cultivation did not influence the content of most of the nutritional beneficial compounds, except the phenolic compounds that were increased with the amounts of pathogens. However, higher amounts of pesticide residues and in many cases also of heavy metals were seen in the conventionally produced crops compared to the organic ones. Animal studies as well as *in vitro* studies showed a clear indication of a beneficial effect of organic food/extracts as compared to conventional ones. Thus, consumption of organic food seems to be positive from a public health point of view, although the reasons are unclear, and synergistic effects between various constituents within the food are likely.

## 1. Introduction—Organic Agriculture Today and in the Future

Organic agriculture is defined today in various ways, although some common principles exist—to not use synthetic fertilizers and chemical pesticides [1]. Some commonly used definitions of organic agriculture are the ones used by FAO—“Organic farming is a holistic production management system which promotes and enhances agro-ecosystem health, including biodiversity, biological cycles, and soil biological activity” [2], IFOAM—“Organic agriculture is a production system that sustains the health of soils ecosystems and people” [3], and USDA—“Organic farming is a production system that excludes the use of synthetically produced fertilizers, biocides, growth regulators, and livestock feed additives such as antibiotics and growth hormones” [4]. Thus, besides the fact that no synthetic fertilizers and chemical pesticides are used in organic farming, there is a general idea that organic farming contributes to improved human and environmental health.

The possibly positive impact on human health as well as on environment is, together with increasing consumer demand, the key reasons why a number of governments have set goals as to how much organic agriculture should increase in their respective countries. Some examples of food policies related to development of organic production are: (i) the Food, Conservation and Energy Act of 2008 in the USA, with a mandatory five-fold funding increase for organic research programs and cost-share assistance for farmers and handlers [5], and (ii) The European Action Plan for Organic farming from 2004, focusing on strengthening information and research as well as improving production standards and streamlining public support [6]. In Africa, the African Union has taken a decision to support and develop frameworks and strategies for organic policies [7].

The mentioned food policies, together with economic subsidies and increased income opportunities for the farmers are part of the explanation for the rapid development of organic agriculture, which is nowadays practiced in more than 160 countries [7]. A total of 37 million hectares of land were grown organically in 2010, which is more than a three-fold increase during the last 10 years, involving 1.6 million producers, although still only 0.9% of the agricultural land is grown organically [7]. From 2009 to 2010, there has for the first time during the last 10 years been a small decrease in the world organic production. However, large increases are still seen in most of the European countries in terms both of increase of agricultural land for organic production, organic market size and *per capita* consumption [7]. However, as to organic producers, more than 80% are found in the developing countries, where the largest share of emerging markets are also present [7]. Still, the main part of the organically produced products in developing countries is exported to developed countries and the main importers of such products are the United States, Germany and France [7]. Due to the fact that we still see an ongoing and steady increase in organic market and production in Europe as well as emerging economies and markets in the developing world, one might expect a continuous increase in the market share of organic products as well as in area of organically certified land around the world for organic production.

Quite a number of studies are available examining the impact on amounts of certain nutritional compounds of organic crop cultivation practices (e.g., [8,9,10]). However, only a few studies (e.g., [11]) have focused on a more holistic and full understanding as to whether organic production influences public health. 

Thus, the aim of the present article is to review and compile results and draw conclusions related to the influence of organic agriculture and crops grown therein on public health. Thereby, conclusions related to food factors influencing public health, nutritional compounds in crops and relations to daily intake as well as significance of organic cultivation on amounts of nutritional compounds are emphasized. Finally, the impact of organic food on public health will be emphasized based on animal, human and *in vitro* studies.

## 2. Important Factors for Public Health—Impact of Nutritional Compounds from Crops

Public health has to a large extent been coupled to lifestyle patterns including dietary changes and selection of daily food [12]. An increasing problem in both developing and developed countries is the commonly reported change in intake patterns resulting in chronic non-communicable diseases (NCDs—non-infectious or transmissible type of diseases), including obesity, cardiovascular diseases, stroke, diabetes *etc.* [12]. Similarly as the intake of sugar and non-essential fats are increasing with the changing lifestyle, nutrient sufficiency in the daily food is decreasing [12]. For a productive life and longevity, sufficient intake of nutrients is essential. Both in the developing and developed world, malnourishment in terms of micronutrients is a large problem, and over three billion people are expected to suffer from such malnourishment [13,14,15]. Thus, besides the diseases mentioned above, resulting from a sedentary life-style with a too high intake of sugar and fat, micronutrient deficiency in the food also contributes to lower productivity, learning disabilities and increased mortality rates [15].

An increased number of recent studies have shown the positive impact of consumption of crop-based foods on human health [13,14,16]. Especially intake of fruits and vegetables has been found important to prevent cardiovascular diseases and obesity [14,16]. Beside fruits, berries and vegetables, other crop-based foods such as e.g., cereals and legumes are also beneficial for human health [17,18]. Several reasons can account for the impact on human health. Firstly, crop-based food is generally low in sugar and fat, especially processed versions, but these food products are also rich in nutritionally beneficial components, e.g., micronutrients and antioxidants or bioactive compounds [14].

Worldwide, nutritional deficiency is known to cause two thirds of all childhood deaths. The main problems in such nutritional deficiency are primarily a too low intake of vitamin A (some carotenoids), iron, and zinc [13,14,15]. However, also other micronutrients such as selenium and iodine are known to be of importance in nutrient deficiency [14], as are other vitamins and bioactive compounds, such as for example vitamin E (tocochromanols) and phenolic compounds [19,20]. Besides causing childhood deaths, deficiency in the mentioned compounds is supposed to contribute to an increased amount of NCDs [21]. Thus, relationships between contents of the mentioned compounds in various crops, influences of organic farming, and relations to public health will be discussed below.

## 3. Content of Compounds in Various Crops Compared to Daily Intake

The recommended daily intakes of various micronutrients differ depending on the specific compound, with high amounts for e.g., calcium (1,000 mg/day), sulphur (850–1,500 mg/day), potassium (3,500 mg/day), and low amounts for e.g., selenium (0.03–0.07 mg/day) and molybdenum (0.05–0.10 mg/day). For the most commonly deficient micronutrients, the recommended daily intake is 0.6 mg/day for vitamin A, 10 mg/day for iron and 10 mg/day for zinc [22,23]. Recommended daily intake for vitamin E is 10 mg/day [22] and for phenols it is 500 mg/day [24].

Recommended daily intake of micronutrients, antioxidants and bioactive compounds can be reached through a proper mixture of suitable food, through intake of multivitamin and/or multimineral pills or through fortification of the food. Recent studies have indicated benefits of natural intake of food with sufficient levels of micronutrients instead of compensation with supplements or pills [25]. Results on smokers even showed an adverse effect on cancer patients with intake of β-carotene and retinol supplementation [26]. Studies on whether fortification of food contributes similarly to health as natural intake of micronutrients, antioxidants and bioactive compounds has been limitedly investigated. To conclude, intake of plant-based food has been shown to contribute to health and a suitable basis for intake of health related compounds [14,16,17,18,26].

While comparing content of various compounds in different crops, presented in a wide array of studies including those on genetic variation, farming practices, and food and nutrition, a large variation is clearly seen (Table 1). Thus, spinach is a good source of iron and zinc while sea buckthorn and rose hips are good sources of vitamin A and E, and black currant is a good source of phenols (Table 1). However, variation is not only seen between various crops but also within specific crops (Table 1). Variation within the crop is the result of variation in genotype, various parts of the crop, between various harvest timing or cultivation places, various colors of the crop or specific breeding activities that have been carried out [8,9,24,27,28,29,30,31,32,33,34,35,36,37,38,39,40,41,42,43,44,45,46,47,48,49,50,51,52,53,54,55,56,57]. Among the latter, genetic modifications of the crop to increase production of a certain compound can be mentioned, e.g., Golden Rice [42,43]. The large variation within a specific crop also results in different requirements for intake of a crop to reach recommended daily intake. Table 2 shows how large a part of the recommended daily intake a certain amount of the crops (with their within crop variation) can contribute as related to certain compounds, when the contribution is calculated from the large variations reported in Table 1. As can be seen from Table 2, total daily requirements of zinc and iron can be reached by consumption of one liter of certain cereals or lentils. However, lentils or cereals are seldom consumed in amounts of one liter a day. Lentils are more commonly consumed in amounts of dL and cereals are mainly eaten as products, e.g., as bread, where one liter of wheat flour is almost enough for two bread loaves, and most of the nutritionally important components are found in the outer layers of the cereal grains, normally not included in the white wheat flour. For vitamin A (pro-vitamin A) or carotenoids, the understanding of human requirements is more complicated. In Table 1 and Table 2 the numbers for various crops are given as total carotenoids. As to requirements, 0.6 mg of vitamin A has been reported [22], although only some of the totally 700 carotenoids (around 50 in the human diet) are precursors of vitamin A [58]. The daily intake of carotenoids has been reported to vary between 9 and 16 mg [59], so in addition to the requirement of 0.6 mg of vitamin A there is most likely requirements of other carotenoids as well as other promoting factors, although the exact amounts are scarcely known. Daily consumptions of 3.3, 2.2 and 1.8 mg β-carotene, lycopene and lutein are reported from an investigation on a Swiss population [47]. As to vitamin E and tocopherols, data in the present paper is built on vitamin E activity, while e.g wheat is known to contain rather high levels of tocotrienols, although without vitamin E activity, but highly relevant for health on other aspects [9]. 

**Table 1:** Variation in content of Iron (Fe; mg/kg), Zinc (Zn; mg/kg), Total Carotenoids (Caro; mg/kg), Vitamin E activity (Vit E; mg/kg) and Total Phenolics (Phe, mg/kg) in a number of representative fruits, berries, vegetables, legumes and cereals.

Crop	Amount of Nutritionally Important Compounds				
	Fe	Zn	Caro	Vit E	Phe
Apple	2–16 [29] *	1–3 [29]		<0.1 [40]	692–1,212 [49]
Orange	1–16 [29]	2–10 [29]		<0.1 [40]	1,080–1,170 [24]
Banana	6–20 [3]°	4–11 [3]°	1–94 [41]	<0.1 [40]	1,060–1,190 [24,50]
Black Currant	1–2 [31]	0.2–0.4 [31]	60–70 [44]		19,200–31,800 [51]
Strawberry	3–7 [32]	1–2 [32]	0.5 [56]	<0.1 [40]	2,230–2,270 [24]
Sea Buckthorn	4–10 [33]	24–38 [33]	120–1,425 [45]	324–452 [37]	21,310–55,380 [60]
Rose Hips	11–118 [34]	7–14 [34]	42–1,024 [46]	110–205 [38]	59,210–122,390 [61]
Carrots	33–37 [35]	24–38 [35]	10–90 [47]	<0.1 [40]	83–85 [24]
Tomato	0.5–1.4 [53]	0.1–0.3 [53]	80–90 [47]	<0.1 [40]	235–239 [24]
Spinach	188–1,255 [54]	40–141 [54]	110–160 [47]		322–329 [24]
Pea	4–5 [36]	2–4 [36]		<0.1 [40]	
Lentils	63–105 [55]	32–39 [55]			
Wheat	18–38 [8]	21–39 [8]	3–4 [57]	6–12 [9]	2,710–3,016 [57]
Rice	1–27 [27]	13–44 [27]	1–37 [43]	17–24 [39]	146–4,222 [48]
Maize	11–34 [28]	14–45 [28]	1–34 [42]		
Oats	45–46 [52]	29–31 [52]			

Note: * Superscripts indicate the reference from where the data is collected, and data collected represents variation in amounts due to genetic variation, farming conditions, sample variation, genetic modifications, *etc*.

## 4. Comparison of Compounds in Organic and Conventional Crops

As mentioned in previous subchapter of this paper, a large variation is seen for nutritionally important compounds in food crops dependent on genotype evaluated, place, year and cultivation practices, part of the crop analyzed *etc.* In a number of previous publications, environmental variation including farming practices have been found as important as genetic variation in determining various components in a crop [62,63,64,65,66,67]. Thus, how the crop is cultivated seems as an important parameter in terms of content of nutritionally important compounds in a crop, and one such change in cultivation practices is organic cultivation instead of conventional. Thus, to verify or reject the hypothesis, that organically grown crops are nutritionally better for human, we will here review the up-to date present results according to influence of organic cultivation on nutritionally important compounds as defined above.

**Table 2:** Daily requirements of some nutritionally important compounds and examples of weight (g) of a certain amount of the various representative crops, and the resulting amount and relative amount in relation to daily requirements of the various nutritionally important compounds (Iron (Fe), Zinc (Zn), Total Carotenoids (Caro), Vitamin E (Vit E) and Total Phenolics (Phe)) from consumption of these weights of the crops.

Crop	Weight (g)	Nutritionally Important Compounds									
		Fe		Zn		Caro		Vit E		Phe	
		Intake (mg)	% of Req	Intake (mg)	% of Req	Intake (mg)	% of Req	Intake (mg)	% of Req	Intake (mg)	% of Req
Daily requirements (mg)		10 [23]		10 [23]		0.6 [22] (Vit A)		10 [22]		500 [24]	
1 Apple	125–150 [68]	0.2–2.4	2.5–24	0.1–0.4	1.2–4.5			<0.02	<0.2	83–181	16–36
1 Orange	175 [68]	0.2–2.8	1.8–28	0.3–1.7	3.5–17			<0.02	<0.2	189–205	37–41
1 Banana	150 [68]	0.2–3.0	2.0–30	0.6–1.6	6.0–16	0.1–14	17–>100	<0.01	<0.1	159–178	31–36
1 L Black Currant berries	500 [68]	0.5–1.0	5.0–10	1.0–2.0	10–20	3.0–3.5	>100			9,600–15,900	>100
1 L Strawberries	500 [68]	1.5–3.5	15–35	0.5–1.0	5–10	0.02	3.3	<0.05	<0.5	1,115–1,135	>100
10 Sea Buckthorn berries	12.5–14.3	0.1–0.1	5.0–14	0.3–0.5	3.0–3.5	1.5–20	>100	4.1–6.5	41–65	266–791	53–>100
10 Rose Hips	14–80	0.1–9.4	15–94	0.1–1.1	1.0–11	0.6–82	>100	1.5–16.4	15–>100	829–9,791	>100
1 Carrots	75–100 [68]	2.5–3.7	25–37	1.8–3.8	18–38	0.7–9	>100	<0.01	<0.1	6.2–8.5	1.2–1.7
1 Tomato	80 [68]	<0.1	<1.1	<0.02	<0.2	6.4–7.2	>100	<0.01	<0.1	18.8–19.1	3.7–3.8
1 L Peas	800 [68]	3.2–4.0	32–40	1.6–3.2	16–32			<0.08	<0.8		
1 L Lentils	800–900 [68]	50–94	>100	25–35	>100						
1 L Wheat	800 [68]	14–30	>100	17–31	>100	2.4–3.2	>100	4.8–9.6	48–96	2,168–2,412	>100
1 L Rice	720-850 [68]	0.7–23	7–>100	9–37	90–>100	0.7–31	>100	12–20	>100	105–3,588	21–>100
1 L Maize	750-800 [68]	8–27	80–>100	10–36	>100	0.7–27	>100				

Notes: * Upper-case numbers indicate number of reference from where the data is collected. Numbers without upper-case numbers are calculated based on data in Table 1. % of Req = Percentage of daily requirement.

Upon reviewing the literature on the content of various compounds in organic and conventional crops, it quickly becomes obvious that conclusions from such comparisons are not easily drawn. One issue in such comparisons is whether the studies were carried out accurately. The importance of comparing crops where the only difference is the cultivation system and not variation in cultivation locations, attributes of the soil, eventual irrigation, whether, crop varieties, harvesting conditions, storage methods *etc*., have been stated by a number of authors [69,70,71,72,73,74]. However, the need of both holistic approaches and reductionist research is commonly pointed out from the organic research society [75], as is the need of research specifically on genotypes suitable for organic production, avoiding studies comparing conventional bred genotypes in conventional and organic systems [76,77]. In our compilation below of results from a wide array of studies related to comparisons of compounds in organic and conventional crops, we have chosen to conclude whether the cited literature stated presence of differences due to farming system applied. We have also chosen to comment on the methodology selected for the comparison in those cases where the studies claim the existence of differences. 

As to variation in micronutrients including iron and zinc, comparisons of contents between organically and conventionally grown crops, have been carried out for almost any crop including e.g., strawberries, tomatoes, haricot beans, cotton, wheat, wheat flour, *etc.* [52,53,54,55,78,79,80,81,82]. The results from these studies shows sometimes a higher content of iron and zinc in the organically produced crops compared to the conventionally grown ones [78,82], however, sometimes the results are the opposite [52,78,79] or there is no statistical difference between the two cultivation systems [53,79,80,81]. The studies showing higher contents of iron and zinc in the organically produced crops [78,82], were not comparative but were based on purchased food from the market. However, some comparative studies have evaluated a number of organic fertilizing strategies and have been able to differentiate certain organic fertilization strategies as resulting in increased levels of iron and zinc in the crops [54,55]. As a whole, this study agrees with previous reviews e.g., [80], concluding that results from various studies are too diverse for a clear recommendation on what cultivation practices should be used for high iron and zinc levels. Other factors, like type of crop, year, place, environment, harvest timing, *etc.*, seem to be of higher importance than organic cultivation. However, as pointed out by Hussain *et* *al*. [8], high content of minerals was found in organically grown wheat of certain genetic background and grown in a certain environment. Thus, it is important to select not only organic farming, but also “the right” genotypes and cultivation environments if high contents of iron and zinc are to be obtained. 

Similarly, a number of studies comparing content of total carotenoids, certain carotenoids and/or vitamin A between various crops grown organic and conventionally have been carried out, e.g., in wheat, green cauliflower, tomatoes, sweet red bell pepper, grapefruit, grapes, apples and carrots [53,56,57,83,84,85,86,87,88,89,90,91,92,93]. The main conclusion from almost all studies correspond with previous reviews within the topic [74], *i.e.* that almost no significant differences in content of carotenoids of any type was found in crops from the two different farming systems [53,56,57,83,84,85,86,87,88,89,90,91,92,93]. Also, no increased plasma levels of lycopene were noted from consumption of organically produced tomatoes compared to conventionally produced ones [94]. In general, weather, environment and genotype seem to play a larger role for the carotenoid content than the farming system [74]. 

Quite a number of publications have evaluated the relationship between tocopherols content as related to organic *versus* conventional farming. A range of different crops have been evaluated in these studies including wheat, barley, rice, strawberries, peach, pears, plums, olives, sunflower, potatoes, fava beans [10,57,93,95,96,97,98,99,100,101]. Generally, the results from most investigations showed no difference in the content of tocopherols in the crops dependent on the farming system applied [10,57,93,97,98,99]. Also, for human health biomarkers, e.g. plasma IgG levels, no evidence of higher value from either of the two farming systems were detected [102]. However, some exceptions as to tocopherol contents were noted in the literature: (i) an overall higher content in total tocochromanol content and a change to a higher tocotrienol/tocopherol ratio were found for organically grown barley as compared to conventionally grown barley [96], (ii) higher contents of α-, and γ-tocopherols in organic plums as compared to conventional plums, especially when grown on natural covered meadow soil [100], and (iii) a higher α-tocopherol content in organic pears as compared to conventionally grown pears [101]. In the three mentioned cases [96,100,101], when differences were found for tocopherols related to the farming system, the experiments had been carried out comparatively, on the same farm or maximum up to 800 m apart, although, field conditions differed somewhat.

Similarly as for the above mentioned micronutrients and bioactive compounds, a large number of studies are available comparing levels of total phenolics as well as of individual phenolic compounds for a large number of crops. Examples of crops evaluated as to various phenolic compounds (or total phenolics) in organic and conventional farming systems are; wheat, maize, oats, potatoes, marionberries, strawberries, blueberries, black currant, peach, pear, apple, kiwi, tomatoes, leaf lettuce, collards, pac choi [92,101,103,104,105,106,107,108,109,110,111,112,113,114,115,116,117,118,119]. Compared with the micronutrients and bioactive compounds discussed above, phenolic compounds seem to be more influenced by the farming system used. Also for phenolics, there are studies with no significant difference in amounts or composition between crops grown conventionally *vs.* organically. However, a number of studies report higher values of various phenolic compounds in organic farming compared to conventional farming systems [101,104,105,107,110,111,112,113,114,116,117,118]. Most of the mentioned studies are comparative, with cultivations either on the same farm or on fields nearby each other. In two studies [113,116], the experiments are carried out on organic and conventional commercial farms. The higher levels of phenolic compounds in organically grown crops have been attributed to the soil organic matter in organically farmed soils. Organic soils are known to have higher microbial biomass and activity, higher biodiversity and more biogeochemical processes [120]. However, the higher levels of phenolic compounds in organic agriculture have also been related to defense mechanisms in the organically grown crops against diseases and pests [104,121].

Thus, to conclude, no clear benefits as to content of the above defined nutritional important compounds could be seen in organically grown crops as compared to conventionally grown crops, with one possible exception, phenolic compounds. Other parameters seem of much higher importance, including selection of genotype, cultivation place, weather and year, harvest time and parts of the crop used. One issue that might be a problem in the evaluations carried out is that mostly cultivars developed for conventional cultivation have been evaluated, and some studies point on the fact that there are clear interaction effects between the selection of plant material and the farming practices [76,77]. Thus, to increase amount of nutritionally important compounds, selection and breeding of suitable genotypes in combination with organic farming might be preferable. Large changes in content of a specific nutritionally important compound has been found in genetically modified crops, e.g., those developed for high β-carotene (pro-vitamin A) content in rice and maize.

## 5. Additional Human Health Related Issues in Crops—Chemicals and Heavy Metals

As mentioned above, organic agriculture does not use synthetic pesticides. Thus, pesticide residues in the food or crop might be one difference between conventional and organic products, which might influence public health. In Germany, a thorough screening of food for active substances of pesticides has been carried out, and a total of 361 active pesticide substances were found, 60.2% of the samples had residues of at least one pesticide and 40,7% had residues of more than one. Table grapes showed the highest numbers of different pesticide residues with 23 substances and in several of the apple, oranges and pear samples 10–12 substances were found [122]. Several studies have indicated that pesticide residues in the food may contribute to the development of cancer, Parkinson’s disease, and endocrine related disorders [123,124,125,126,127]. 

Urinary biomonitoring has been used to evaluate dietary organophosphorus pesticide exposure among children, and showed that a substitution into organic food for five consecutive days decreased two specific metabolites, malathion and chlorpyrifor, to undetectable levels [128]. Furthermore, children who consumed exclusively organic produce showed no measurable pesticide metabolites [129]. 

As to heavy metals in crops and crop-based food, some literature states that the contents do not differ significantly as related to if the applied farming system has been organic or conventional [130]. However, when screening the literature and investigations on various crops and crop-based food from the two systems, there is a number of studies showing higher contents of heavy metals and especially cadmium from conventional grown products than from organic ones e.g., [131,132,133,134,135,136,137]. A few studies also report on higher cadmium (Cd) and lead (Pb) levels in organic tomatoes, but lower levels of Cu as compared to conventional ones [138], and higher levels of nickel, Pb and zinc (Zn) in organic wheat, as compared to conventional [131]. One explanation for higher levels of heavy metals in conventional than organic crops and crops based food might be the higher levels of Cd, copper and Zn in inorganic fertilizers that commonly have been used, although presence of heavy metals in conventional fertilizers has been reduced during recent years [139]. Similarly as for the nutritional compounds discussed above, a large variation in heavy metal accumulation among genotypes, also when organically grown, have been reported [140].

## 6. Health Biomarkers in Whole Organisms and *in vitro* Studies

In addition to estimations of the content of individual compounds in different organic *vs.* conventionally cultivated crops, investigations have been performed on the effects of health biomarkers in whole organisms, or effects studied in *in vitro* systems. These experiments may represent a more holistic approach and also comprise synergistic effects between different compounds, which otherwise might be difficult to investigate and evaluate. In different animal experiments, investigated effects of organic or conventional feed have mainly concerned two areas; fertility/reproduction and the immune system. Early studies, starting as early as 1926, investigated the outcome when organic fertilizers were compared with mineral, and found differences in growth rate (rats, two studies), pup survival rate (mice), reproduction (chicken), and fertility (bulls, rabbits), though three studies found no differences in growth rate or fertility [141]. Similarly, more recent studies have found differences on animals fed with organic *versus* conventionally grown feed, although in some studies no significant differences were observed. Positive results from organic feed were found for reproductive performance in rabbits [142], immune status in rats and chicken [143,144], resulting in stimulated proliferation of lymphocytes in rats if fed with protein shortage [133], higher levels of α-tocopherol and immunoglobulin G in blood serum [145], higher splenocyte proliferation in male rats [146], increase in regulatory T cells in mice [147], enhanced immune reactivity, a stronger reaction to the immune challenge as well as a slightly stronger “catch-up growth” after the challenge in chicken [143]. Recently also the well characterized fruit fly, *Drosophila* *melanogaster*, was used in experiments with organic and conventional feed. Flies raised on diets made from organic produce (bananas, potatoes, raisins, soy beans) had greater fertility and longevity than those raised on conventional produce [148].

Interestingly, several studies with mice, rats, rabbits and hen, have reported that animals can discriminate between organic and conventional feed in food preference tests and prefer organic produce [141,149,150].

Conducting long-term human studies has some obvious difficulties. Often preference for organically produced food is associated with a healthy and active life style [151], which has recently been emphasized in a follow-up study on the large Nutrinet-Santé cohort, comprising 54,311 adults [152]. The study concluded that consumers of organic products were more highly educated, more physically active, exhibited dietary patterns including more plant food and less sweet and alcoholic beverages, as compared with those not interested in organic products [152]. Most human short-term intervention studies have shown no significant difference for people eating conventional or organic food. Thus, no significant difference was found in total antioxidant activity, serum LDL oxidation or total phenol content in humans consuming organic *versus* conventional Cabernet Sauvignon wine [153], in serum glucose, triacylglycerol and uric acid, LDL antioxidant capacity and lymphocyte damage in humans consuming apples [154], polyphenol and carotenoid concentration in humans consuming apples and carrots, and LDL oxidation and some immune factors in humans consuming carrots [155,156]. However, urinary excretions of quercetin and kaempferol were higher after consumption of organic diet [157], and consumption of organic dairy products, but not organic diet, reduced risk of eczema among children [158]. Children eating an antroposophic diet in an observational study, as compared with conventional diet, had lower body weight and exhibited fewer allergies [159]. The discrepancy between the outcome of the animal studies, showing a rather wide array of positive effects of organic food, and the short-term human studies, only showing a few positive effects, has resulted in questions related to planning and performance of human studies. Many studies have tried to find reliable biomarkers for health, and recent animal feeding studies have had as an aim to define disease risk and health effects' biomarkers usable for future human studies [145,160]. However, still there is only a limited number of human studies available having investigated the effects of consumption of organic food on health, disease risks’ and health promoting compounds, and the development of reliable biomarkers to be used in such studies are still in its infancy [161,162]. Therefore, as we today routinely accept animal experiments for assessing the risk of toxic food substances, to study absorption, metabolism and function of nutrients, as well as investigating beneficial effects of components in food, it seems reasonable that the positive results of the animal feeding experiments with organic food can be considered as a good indication of that there might be health benefits of consumption of organic food also for humans. 

*In vitro* studies have shown that fruit and berry extracts inhibit cancer cell proliferation [163]. Thus, constituents from fruits and especially berries seem to affect cancer progression cellular processes [164]. Also, extracts from strawberries were found to inhibit cancer cell proliferation rate and extracts from organic strawberries to a higher degree than extracts from conventionally produced strawberries [165]. Despite these findings, most literature indicates that there is no proof of specific compounds, nor of high antioxidant activity contributing to anticancer activity [166]. This means that the exact mechanisms behind the anticancer activities seen in *in vitro* studies from berries are still not known, although further *in vitro* studies and models might contribute to increased understanding of mechanisms [167]. 

## 7. Conclusions—Do Organically Produced Crops Contribute to Human Health?

**Figure 1:**
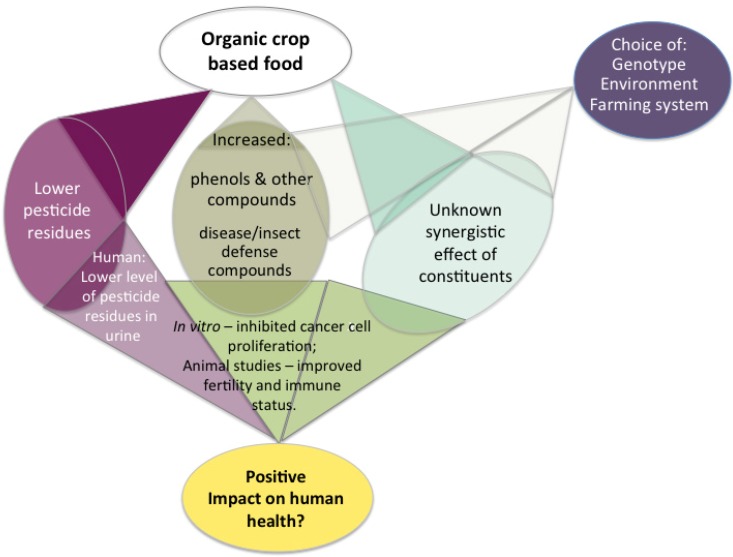
Interactive model showing proof of concept for interaction of different factors resulting in a possible positive impact on human health by organic crop based food.

Both animal studies and *in vitro* studies clearly indicate the benefits of consumption of organically produced food instead of that conventionally produced. Investigations on humans are scarce and only few of those performed can confirm positive public health benefits while consuming organic food. However, animal experiments are today routinely used to assess impact on humans in various other aspects and thus, the positive effects on animal from consumption of organically produced food can be regarded as an indication of positive effects also on humans. The reasons why organically produced food contributes to public health are unclear, as specific high amounts of nutritionally high value compounds with high antioxidant capacity does not seem to be the key for improved public health from organic food consumption. Instead synergistic effects of several constituents might be the back-ground for the possible positive effects of organic food, as well as absence of pesticide residues. The present review also did not find higher contents of nutritional beneficial compounds, with the exception of phenolic compounds, as an answer to increased amounts of pathogens, in organically cultivated crops than in conventionally cultivated ones. Some previous studies have indicated a higher vitamin C content in organically produced crops as compared to conventionally ones [107], as a response to increased amounts of pathogens [107]. Vitamin C might be part of the synergistic constituents contributing to prevention against oxidation of other compounds although vitamin C in itself was not found to protect against proliferation of cancer cells [149]. Extremely large variation of nutritional beneficial compounds was found in crops due to various reasons, and parameters contributing to the variation were: genotypes, farming conditions, environment, harvest time, crop part and genetic modifications of the crop. The highest values of specific nutritional compound were seen in specific genotypes and high increases were seen for genetically modified crops. These crops with very high values of certain compounds could be of relevance for cultivation and production of food in areas with a large deficit in that specific compound in the diet. For a general improvement in public health, it however, seems more relevant to focus on combining a “right” genotype with a “right” farming system, were organic farming could be an alternative. From this “right” system, a crop based food should then be obtained with the “right” cocktail of constituents to obtain the synergistic effect that was reported from organically produced fruits and berries as related to cancer cell proliferation. The low amounts of pesticide residues and heavy metals reported in organically produced crops might be one part of the reported bases for an anticancer effect of organic food. The reasons for an eventual positive effect of consumption of organic crop-based foods on public health are summarized in Figure 1. Besides the impact on public health through possible positive effects of consumption of organic food, public health might also be influenced through organic cultivation due to its environmental effects, which are not discussed in the present review.
